# Migration of rice planthoppers and their vectored re-emerging and novel rice viruses in East Asia

**DOI:** 10.3389/fmicb.2013.00309

**Published:** 2013-10-28

**Authors:** Akira Otuka

**Affiliations:** Kyushu Okinawa Agricultural Research Center, National Agriculture and Food Research OrganizationKumamoto, Japan

**Keywords:** rice planthoppers, migration, trajectory analysis, entomological radar, virus disease

## Abstract

This review examines recent studies of the migration of three rice planthoppers, *Laodelphax striatellus, Sogatella furcifera*, and *Nilaparvata lugens*, in East Asia. *Laodelphax striatellus *has recently broken out in Jiangsu province, eastern China. The population density in the province started to increase in the early 2000s and peaked in 2004. In 2005, *Rice stripe virus* (RSV) viruliferous rate of *L. striatellus* peaked at 31.3%. Since then, rice stripe disease spread severely across the whole province. Due to the migration of the RSV vectors, the rice stripe disease spread to neighboring countries Japan and Korea. An overseas migration of *L. striatellus* that occurred in 2008 was analyzed, when a slow-moving cold vortex, a type of low pressure system, reached western Japan from Jiangsu, carrying the insects into Japan. Subsequently the rice stripe diseases struck these areas in Japan severely. In Korea, similar situations occurred in 2009, 2011, and 2012. Their migration sources were also estimated to be in Jiangsu by backward trajectory analysis. *Rice black-streaked dwarf virus*, whose vector is *L. striatellus*, has recently re-emerged in eastern China, and the evidence for overseas migrations of the virus, just like the RSV’s migrations, has been given. A method of predicting the overseas migration of *L. striatellus* has been developed by Japanese, Chinese, and Korean institutes. An evaluation of the prediction showed that this method properly predicted migration events that occurred in East Asia from 2008 to 2011. Southern rice black-streaked dwarf virus (SRBSDV) was first found in Guangdong province. Its vector is *S. furcifera*. An outbreak of SRBSDV occurred in southern China in 2009 and spread to Vietnam the same year. This disease and virus were also found in Japan in 2010. The epidemic triggered many migration studies to investigate concrete spring-summer migration routes in China, and the addition of migration sources for early arrivals in Guangdong and Guangxi have been proposed. *Nilaparvata lugens* is also an important insect pest of rice. Its migration situations on the Indochina peninsula and return migrations in China are discussed.

## INTRODUCTION

Some viruses in economic plants spread as they are carried by their invertebrate vectors (Matthews, 1991), and sometimes even migrate overseas. Rice planthoppers, major rice pests, and their vectored rice viruses are examples. Rice planthoppers consist of three species: the small brown planthopper, *Laodelphax striatellus* (Fallén); the white-backed planthopper, *Sogatella furcifera* (Horváth), and the brown planthopper, *Nilaparvata lugens* (Stål; Hemiptera: Delphacidae). Currently, rice planthoppers have been causing various problems in East Asia. An outbreak of *N. lugens* occurred in China, Korea, and Japan in 2005 (Cheng and Zhu, 2006). Major reasons for the outbreak included favorable weather conditions and insecticide resistance of the vector (Cheng and Zhu, 2006; Matsumura et al., 2008). *N. lugens* and its rice viral diseases also severely damaged rice in southern Vietnam in 2006–2007, and the Vietnamese government temporarily stopped the country’s rice exports, which affected the world rice market (Chien et al., 2007). *S. furcifera* has caused a novel virus disease in southern China since the early 2000s, and the disease later spread to wide paddy areas in China, northern to central Vietnam, and Japan (Zhou et al., 2010; Hoang et al., 2011; Matsumura and Sakai, 2011). The density of *L. striatellus* in eastern China rapidly increased in the mid 2000s, and an outbreak of rice strip disease occurred (Zhou, 2010). Rice strip disease has spread to Japan and Korea from 2008 to the present (Otuka et al., 2010; Lee et al., 2012). Meanwhile, local plant protection institutes in each country conducted intensive surveys and took effective control measures against the vectors and diseases (Zhou, 2010).

Recent outbreaks of rice planthoppers and related virus diseases that occurred in East Asia were closely related to the vectors’ migration. This review, therefore, examines the recent development of migration studies of rice planthoppers in East Asia, and presents a vivid image of the dispersion of viruses vectored by the insects. The review consists of three parts, covering the migration of *L. striatellus*, the migration of tropical rice planthoppers *S. furcifera *and *N. lugens*, and a discussion.

## Laodelphax striatellus

**FIGURE 1:**
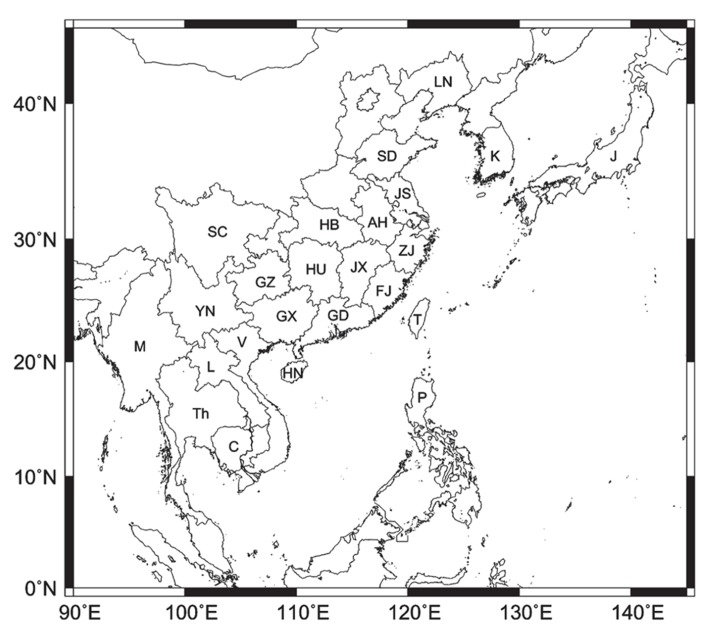
**Locations of Chinese provinces and East Asian countries of interest**. Abbreviations LN, SD, JS, AH, ZJ, HB, JX, FJ, HU, GD, GZ, GX, HN, SC, T, P, V, C, L, Th, M, K, and J indicate Liaoning, Shandong, Jiangsu, Anhui, Zhejiang, Hubei, Jiangxi, Fujian, Hunan, Guangdong, Guizhou, Guangxi, Hainan, Sichuan, Taiwan, the Philippines, Vietnam, Cambodia, Laos, Thailand, Myanmar, South Korea, and Japan, respectively.

This species is widely distributed in East Asia, including Japan, Korea, and China, and transmits *Rice stripe virus *(RSV) to rice plants in persistent and transovarial manners (Falk and Tsai, 1998). Since *L. striatellus* is able to overwinter at mid-latitudes, including all areas in Japan, outbreaks of rice stripe disease in Japan had been mostly believed to be caused by domestic populations until 2008, when there was an overseas mass migration of *L. striatellus *in early June in western Japan followed by an outbreak of rice stripe disease (Otuka et al., 2010). The migration source was thought to be Jiangsu province, eastern China (**Figure 1**; Otuka et al., 2010). A set of events related to the migration, therefore, had started in China.

### RECENT SITUATION IN EASTERN CHINA

The rice-wheat or -barley double-cropping system is used in Jiangsu. Indica hybrid rice was introduced there in the late 1970s in order to increase rice yield, and by the mid-1980s the area of hybrid rice accounted for a third of the total rice area (Gu et al., 2005; Sogawa, 2005). Since *L. striatellus *cannot effectively multiply on hybrid rice compared with japonica rice (Liu et al., 2007), rice stripe disease was not a problem until the end of the 1990s (Sogawa, 2005). In the late 1990s, the ratio of japonica rice started to increase in the province, because japonica varieties of good taste were more profitable in the market than indica hybrid rice varieties (Gu et al., 2005); by 2002 the area of japonica rice exceeded 80% of the total (Sogawa, 2005). However, major japonica varieties used in Jiangsu, e.g., Wuyujing 3 and Wuyunjing 7, were susceptible to both *L. striatellus* and RSV (Yang et al., 2002; Gu et al., 2005). The prevalence of susceptible varieties was the first factor for the outbreak of the insect. Secondly, early rice seeding and early transplanting became popular especially in the middle and northern parts of the province, in order to obtain high and stable yields, making the best use of high temperatures in summer (Yang et al., 2002). This resulted in the overlap of wheat harvesting and rice seedling, making it much easier for the host transfer of *L. striatellus* long-winged adults (Yang et al., 2002). Thirdly, the practice of direct seeding of wheat and barley without plowing after rice harvesting spread in Jiangsu in order to reduce labor. In these fields, the insects transferred easily from rice to wheat or barley (Yang et al., 2002). These three major factors helped the insects rapidly multiply in Jiangsu (Sogawa, 2005). The viruliferous rate of the first generation in Jiangsu peaked at 31.3% in 2005 (Zhou, 2010). Rice stripe disease consequently spread across the entire province. The occurrence area of this disease in paddy fields peaked at 1.57 million ha (79% of the total paddy area) in Jiangsu in 2004 (Zhou, 2010). The use of chemicals to control viruliferous vector insects has been one of the main measures used. An insecticide, imidacloprid or a Chinese product named *bichonglin*, was recommended to spray (Gu et al., 2005; Xi et al., 2005; Xian et al., 2005), but it was applied on average at least five times in a single summer crop (Zhou, 2010). These intensive uses resulted in the development of resistance against the insecticide (Ma et al., 2007; Otuka et al., 2010; Sanada-Morimura et al., 2011).

*L. striatellus* and rice stripe disease in Jiangsu have been managed as follows. One of the main methods of controlling rice stripe disease was to introduce rice cultivars resistant to the virus (Iizuka, 1989; Zhou, 2010). When an epidemic of rice stripe disease occurred in the 1980s in the Kanto district of eastern Japan, cultivars resistant to the virus were introduced and the epidemic quickly ceased (Iizuka, 1989), indicating the effectiveness of resistance cultivars. Other methods to control the disease included chemical control of the vectors, introduction of a gap between wheat and rice cultivations (Kiritani, 1983; Wang et al., 2008; Zhou, 2010), covering of rice seedlings with an insect-proof mesh, and plowing before the seeding of wheat and barley in autumn (Zhou, 2010; Zhu, 2012). All the measures taken in Jiangsu are a standard way to control the pest, and the importance of them has been re-recognized there. With these integrated measures, the RSV viruliferous rate in Jiangsu has decreased to a low level of 3% in 2012 (Zhu, 2012).

### OVERSEAS MASS MIGRATION OF *L. steriatellus*

The 2008 overseas mass migration of *L. steriatellus *occurred in western Japan under these circumstances in China. Large catches of *L. steriatellus* adults by a net trap (63 insects) or a suction trap (106) were recorded on June 5, 2008, simultaneously at two different sites 100 km apart on Kyushu Island (Otuka et al., 2010). In order to allocate the possible source of the migration, a backward trajectory analysis that traced air parcels backward from points over the trap sites was conducted, and the trajectories reached Jiangsu in about 24–36 h, suggesting Jiangsu may have been the source (Otuka et al., 2010). Immigrants were collected from rice fields in immigrated areas in Japan within 2–4 days after the migration. Insects in Jiangsu province were also collected at the end of September to early October 2008. These insects’ resistance to insecticides was tested, and both the immigrants and the Jiangsu population showed resistance against imidacloprid with high LD_50_ values in the topical application method, whereas Japanese domestic populations collected in Kyushu before the 2008 migration event showed susceptibility to the same insecticide (Otuka et al., 2010). In addition, the RSV viruliferous rates of the immigrant populations were reported to be higher (9.2–11.5%) than those of the domestic populations (2.9–4.0%) by enzyme-linked immunosorbent assays (Nakagawa and Mizobe, 2010; Otuka et al., 2010), indicating supportive evidence for the overseas migration.

An outbreak of rice stripe disease subsequently occurred on the western coast of the Kyushu and Chugoku districts (Ohtsu et al., 2009; Otuka et al., 2010; Nakagawa and Mizobe, 2010). The total occurrence area of rice stripe disease in Nagasaki prefecture in 2008 was 10,720 ha, which was a 126% increase over the previous year (MAFF, 2009). All these analytical results suggested that *L. steriatellus* migrated from Jiangsu to western Japan and caused the outbreak of rice stripe disease.

### IN KOREA

Responding to the mass migration to Japan, Korean scientists quickly set up a monitoring network of 13 net traps (10 m high above the ground) for *L. striatellus *along their western coast in May 2009. A similar mass immigration occurred from May 30 to June 1, 2009 (Choi et al., 2010; Otuka et al., 2012a). The catch numbers at Sinan and Taean, located along the western coast of the Korean peninsula, were 819 and 963, respectively, about 10 times larger than those in the previous Japanese case. Based on the backward trajectory analysis, a possible migration source for the Korean case was found to be Jiangsu (Otuka, 2009). The RSV viruliferous rate of *L. striatellus* was 5.3%, and the occurrence area of rice stripe disease, located along the western coastal areas, was 21,500 ha in 2009 (Lee et al., 2012). Similar migration events occurred also in Taean, Gunsan, and Buan in 2011, and in Taean in 2012 (Jeong et al., 2012; Lee et al., 2012). According to surface weather maps for the times when these migration cases occurred, low-pressure systems over Bohai Sea in 2009 and 2011, and a high pressure system over the southern Yellow Sea in 2012, caused southwesterly winds that might have carried the insects to Korea.

### CHARACTERISTICS OF THE OVERSEAS MIGRATION

The frequency of possible overseas migration of *L. striatellus* into the northern Kyushu district in relation to weather conditions was analyzed (Syobu et al., 2011). The investigation covered the 10-year period from May 21, 2000, to June 10, 2009. One peak trap catch was recorded on May 27–28, 2006, and was associated with strong westerly winds at 850 and 925 hPa levels to the south of a cold vortex that passed over the southern part of the Korean peninsula. The backward trajectory analysis suggested Jiangsu, China, as a possible migration source. Another case was the event in 2008 mentioned above. No immigration was found in Japan from 2010 to 2011 (Otuka et al., 2012a). Thus, in total two overseas migrations of *L. striatellus* may have occurred in 12 years in Japan.

On the other hand, three overseas migration events may have occurred in Korea form 2009 to 2012. In addition, Lee et al. (2012) reported that severe damage to rice by rice stripe disease occurred in western areas in 2001, 2007, and 2008, but it is not certain whether these damages were caused by local *L. striatellus* populations or overseas immigrants. However, a migration was predicted in 2008 by Figure 6a in Otuka et al. (2012a), and the predicted areas in southwestern Korea matched the damaged areas: Sinan, Jindo, Haenam, and Wando (Kim et al., 2009; Lee et al., 2012). Therefore, the 2008 case may be one of the overseas migrations. Thus, the frequency of a possible overseas migration in Korea would be four times in 5 years (2008 to 2012), which is higher than that in Japan. The difference in the event frequency is attributable to the location of the destination from the source. Korea is northeast of Jiangsu, whereas Japan is west of Jiangsu. In the middle latitudes, southwesterly winds blow frequently to the south of a low pressure system. The low-level jet stream over the East China Sea carrying various insects is a typical example (Seino et al., 1987). In contrast, sustaining westerly winds by a cold vortex seems less frequent (Syobu et al., 2011). Therefore, it can be said that Korea is unfortunately located in a migration-preferred direction from the source area.

The distance between the source and the destination might have affected the number of immigrants. The distance between western Japan and the coastal line of Jiangsu province is about 750 km, whereas that between the Korean peninsula and Jiangsu is about 500 km. The catch numbers in the net trap in Korea in 2009 were more than ten times larger than those in Japan. Moreover, the distribution of the immigrated areas in Japan and Korea is of interest. All estimated immigrated areas were confined to coastal regions (Ohtsu et al., 2009; Nakagawa and Mizobe, 2010; Lee et al., 2012). For example, in 2008 rice stripe disease occurred heavily in Nagasaki prefecture, located in the westernmost part of Kyushu Island, but no epidemic of the disease was reported in a neighboring prefecture, Saga (Ohtsu et al., 2009). In Yamaguchi prefecture, the outbreak of the disease happened mainly in the coastal areas (Nakagawa and Mizobe, 2010). Similarly, the disease’s recent occurrence area in Korea was distributed along the western coastal line. These facts imply that *L. striatellus *invaded these areas by flying at low altitudes, because an outbreak of the disease could have occurred inland if the insects had flown at altitudes high enough to cross the mountains.

### DOMESTIC DISPERSION AND MIGRATION IN CHINA

When the outbreak of *L. striatellus* and rice stripe disease spread to all of Jiangsu around 2004, the neighboring provinces of Zhejiang, Anhui, Shanghai, Shandong, and Hubei started to suffer similar problems (Wang et al., 2008). In Zhejiang province, the disease spread rapidly southward, from the northern to centraland eastern regions, with an increasing incidence each year from 2003 to 2006 (Wang et al., 2008). By 2006, the disease was severe in the northernmost parts of the province. The timing of the epidemic in Zhejiang province was just after the outbreak in Jiangsu. Increasing populations of RSV viruliferous vectors early in the season were clearly the primary source of the epidemic (Wang et al., 2008). Wang et al. (2008) did not discuss the source of the vectors. However, the situation in Zhejiang province and that in Jiangsu province suggest that the migration of viruliferous *L. striatellus* from Jiangsu could be, at least partly, the cause of the epidemic. More clearly, domestic migrations of the vectors were recently investigated in Zhejiang, Anhui, and Shandong provinces (Wan et al., 2011; Zhang et al., 2011; He et al., 2012). For example, a large immigration of *L. striatellus* in Jining, southern Shandong province, north of Jiangsu province, was observed by a light trap in the late night of 7 June, 2009, and backward trajectories suggested that a possible migration source was northern Jiangsu province (Zhang et al., 2011). They also studied forward trajectories for an emigration peak on 15 June, 2010, and the trajectories reached Liaoning province, suggesting a domestic overseas migration (Zhang et al., 2011). Similarly, possible migration from Jiangsu province to Liaoning province, a kind of overseas migration, has also been suggested (Zhou and Cheng, 2012).

### DIVERSITY OF RSV

The vector migrates a long distance, and RSV does so as well. Therefore, the distribution of a viral population may be affected by the host’s migration. The genetic diversity of the virus has been studied in East Asia (Wei et al., 2009; Jonson et al., 2009; Sakai et al., 2011). These studies included phylogenetic analyses of the nucleotide sequences of nucleocapsid protein (N) and RNA3 intergenicregion (IR3) or the whole sequence of RNA3 of RSV isolates collected in Japan (collection years 2008 and 2009), eastern China (1997–2004), and western Korea (2007–2008). The results showed that RSV isolates in China were divided two types, labeled type I and II. The isolates from eastern China including Jiangsu consisted of type I, and those from Yunnan province, southern China, formed type II. Isolates from the Kyushu district of western Japan and most of the isolates from western Korea belonged to type I. Additionally, isolates from the Kanto district, eastern Japan, and one isolate from Korea formed another subtype (J-K subtype in Sakai et al., 2011, or type II in Jonson et al., 2009). The distance between the Kyushu and Kanto districts is about 1,000 km, and the Kanto district is far from Jiangsu. These studies suggested that the RSV populations in the Kyushu district, Korea, and eastern China are indistinguishable from each other, and that the migration of *L. striatellus* from eastern China to Japan and Korea may have affected the structure of the RSV population in East Asia.

### RICE BLACK-STREAKED DWARF VIRUS

Rice black-streaked dwarf disease caused by *Rice black-streaked dwarf virus* (genus *Fujivirus*; RBSDV) emerged in late *japonica* rice in Zhejiang province, eastern China in 1989, and expanded in the 1990s, having four major outbreaks in 1992, 1996, 1997 and 1998 (Wang et al., 2009). The epidemic of the disease on late *japonica* rice in Zhejiang province continued in the 2000s, and the total affected area increased from 26,000 ha in 2000 to 64,640 ha in 2005, spreading from eastern part to central and southern parts of the province (Wang et al., 2009). The virus has been detected in almost all the area of the province from 2008 to 2011(Wu et al., 2013). As the density of the vector *L. striatellus* in Jiangsu province, a northern neighboring province of Zhejiang, increased in the 2000s as described above, the occurrence of rice black-streaked dwarf disease also increased from 20,500 ha in 2007 to 33,300 ha in 2009, and the paddy area of complete yield loss in the province reached to 2,000 ha in 2008 (Lan et al., 2012). When overseas mass migrations from Jiangsu province to South Korea possibly happened, the RBSDV viruliferous rate of *L. striatellus* caught in net traps along the western coast of the Korean peninsula in early June 2009 and 2011 were found to be 3.1 and 4.4 percent, respectively (Kim, 2009; Jeong et al., 2012), indicating that migrations of both RSV and RBSDV likely occurred due to the vectors’ movement over the sea. No vector immigrant with co-infection of RSV and RBSDV in Korea has been found so far (Kim, 2009; Jeong et al., 2012). Since no resistant gene of rice for RBSDV has been found, vector control by chemicals in early susceptible stages of rice plants, and the use of an insect proof net for rice seedlings are major effective measures of disease control (Lan et al., 2012).

### PREDICTION OF MIGRATION OF *L. striatellus*

A method has been developed to predict the overseas migration in East Asia (Otuka et al., 2012a). The source of the migration is assumed to be Jiangsu, China. The method consists of two steps: prediction of the emergence of the first generation of *L. striatellus* (the first step), and simulation of the migration route during a predicted emigration period (the second step; Otuka et al., 2012a).

The prediction of emergence is performed by calculating the daily increase in effective accumulative temperature (EAT) for *L. striatellus* starting from the first day of each year. The EAT is calculated with daily minimum and maximum temperatures in Dongtai, a city in central Jiangsu, and the data are obtained through the Internet in real time. Growth parameters of the insect for the EAT calculation, such as low-limit temperature and growth-stop temperature, are those estimated for Japanese populations. The EAT value is updated daily, and a presumed increase is added until the predicted value exceeds a pre-defined threshold. The threshold for the emergence was previously determined by analyzing past migration events. The migration prediction period is 9 days, starting from the predicted emergence day – 3 days (prediction error), and ending on the emergence day + 5 days (pre-emigration period of 2 days + prediction error). This is the prediction period.

Migration is then predicted during the prediction period (the second step). In the prediction model, insects take off at dusk and dawn then fly upward at a speed of 0.2 m/s for an hour to make use of upper winds favorable to migration. There is no such observation for *L. striatellus* so far, but there is a radar observation of *N. lugens* flying upward in autumn in Jiangsu (Riley et al., 1991). During migration the insects move at the velocity of the wind, which is forecasted by a weather prediction model. The migration lasts 24 h. The relative aerial density of the insects in the lowest calculation layer is calculated on the basis of the number of insects in each grid cell, and is drawn on a map. By this prediction, the areas and timing of immigration in Japan and Korea are presented. The prediction method was evaluated against the past migration events occurring in Japan and Korea, and the prediction had a 90% accuracy rate (Otuka et al., 2012a). Now the method is implemented and operational in JPP-NET (), database service provided by the Japan Plant Protection Association, Tokyo.

## *Sogatella furcifera* AND *Nilaparvata lugens*

### EPIDEMIC OF SRBSDV AND MIGRATION OF ITS VECTOR

*S. furcifera* is a vector of SRBSDV that causes stunting, leaf darkening, and small enations on the stem and leaf back of rice plants (Zhou et al., 2008, 2013). This disease was first recognized in 2001 in Guangdong province, southern China, and in the next few years spread gradually to Guangxi, Hainan, Hunan, and Jiangxi provinces, but its infected hill ratio was low, 1% or less (Zhou et al., 2008, 2010). In 2009, however, an epidemic of the disease occurred in Guangdong, Guangxi, Hainan, Hunan, Hubei, Jiangxi, Fujian, Zhejiang, and Anhui provinces; the total infected paddy area was about 400,000 ha, and a paddy area of 6,700 ha suffereda complete yield loss (Zhou et al., 2010; Zhao et al., 2011b). In the same year, an outbreak also occurred in Vietnam, where 19 central to northern provinces suffered from the disease (Hoang et al., 2011). In 2010, the infested area in China increased to more than 1.3 million ha in 13 provinces (Zhao et al., 2011b). In August 2010, the disease was first detected in forage rice in western Japan (Matsumura and Sakai, 2011). The virus was detected also across all of Zhejiang province by 2011 (Wu et al., 2013). The rapid spread of the virus throughout East Asia attracted scientists’ renewed attention to the migration of the vector, *S. furcifera.*

### EARLY IMMIGRATION IN GUANGDONG AND GUANGXI

Since Guangdong and Guangxi are possible sources of *S. furcifera* and *N. lugens* immigrants in more northern paddy areas in China (Cheng et al., 1979; NCRG, 1981), early immigrations from April to early May in these areas and their sources are important. Recent trajectory analyses with the simulation model HYSPLIT (Draxler and Hess, 1998) in these areas indicated that possible sources of the early migrations of *S. furcifera* and *N. lugens* were Hainan province, northern and central Vietnam, and southern Laos (Qi et al., 2010a, 2011; Shen et al., 2011b; Wang et al., 2011b; Jiang et al., 2012). The winter-spring rice crop along the coast of central Vietnam is earlier than that in northern Vietnam where the main emigration begins in May (Otuka et al., 2008; Zhai and Chen, 2011), and rice plants in the central region mature in April, which makes them a possible source (Shen et al., 2011b). In addition, SRBSDV in Vietnam in 2009 was distributed in the central regions as well as in the northern delta, suggesting that there was viral dispersion to central Vietnam from southern China, where SRBSDV originated (Hoang et al., 2011). Laos’s border with Vietnam is mountainous, providing a possible barrier to migrations heading northeast. Chinese hybrid rice susceptible to *S. furcifera* is popular in northern Vietnam (Hoang et al., 2011), while sticky rice is popular in Laos. Information on the occurrence of rice planthoppers in spring in southern Laos is limited. Hence, it may be premature to conclude that Laos is a source of the early migration, and further investigation of rice planthoppers’ migration and the occurrence of SRBSDV in Laos is necessary. It is likely that both the central and northern parts of Vietnam, as well as southern Hainan province in China, are source areas for the early immigration to Guangdong and Guangxi (Zhai et al., 2011).

It is generally believed that the East Asian populations of *S. furcifera* and *N. lugens* overwinter in Vietnam and southern Hainan province, and that in spring they migrate northeastward to eastern China, Japan, and Korea utilizing southwesterly monsoons, then migrate southward back to overwintering areas in autumn (Kisimoto, 1976; Cheng et al., 1979; NCRG, 1981). These tropical rice planthoppers of the Red River Delta in northern Vietnam have been recognized as a main source for the East Asian population, since *N. lugens *shifted from biotype 1 to biotype 2 synchronously in northern Vietnam, China, and Japan from the late 1980s to the beginning of the 1990s (Sogawa, 1992). Now most scientists who study rice insect pests believe the rice planthoppers originally migrated from northern Vietnam and southern Hainan (Zhai et al., 2011). The recent studies on the early migrations in Guangdong and Guangxi presented a modification of the initial belief about migration.

Regarding the epidemic of SRBSDV in China, migrations of *S. furcifera* in 2009 and 2010 in the northeastern paddy areas between June and July have been investigated as well (Zheng et al., 2011; Zhao et al., 2011a,b; Diao et al., 2012). Immigration sites were located in central Zhejiang, southern Anhui, and southern Jiangxi provinces. Their possible sources were estimated to be Guangdong, eastern Guangxi, southern Jiangxi, and southern Fujian provinces for the Zhejiang case; Jiangxi and Hunan provinces for the Anhui case; and mainly Guangdong province for the Jiangxi case. Additionally, source areas for *S. furcifera* immigrations in the Kyushu district, western Japan, in June and July, were estimated to be mostly Fujian (Otuka et al., 2005). On the other hand, source areas for *S. furcifera* immigrants in southern Fujian in April to May from 2007 to 2010 were estimated to be Guangdong and Hainan (Shen et al., 2011a). Possible immigration sources in April to May in western Taiwan were also estimated to be Guangdong, Hainan, and the Philippines (Huang et al., 2010).

### YUNNAN PROVINCE AND INDOCHINA PENINSULA

Yunnan province is located east of Myanmar, north of Laos and Vietnam, west of Guangxi and Guizhou provinces, and south of Sichuan province (**Figure 1**). Temperatures in winter and spring in the two-crop rice area in Yunnan are relatively high due to the low latitude, and small numbers of *N. lugens* and *S. furcifera* can overwinter on rice seedlings and ratoons (Shen, 2010). But immigrants in spring cause most of the major damage (Shen, 2010). Sources of *S. furcifera*’s large immigrations in April to early May in Yunnan were estimated to be mainly Myanmar, and immigrations in mid-May were thought to be from northern Vietnam (Shen et al., 2011c). In Myanmar, new irrigation systems have been developed recently and the winter-spring rice crop in the dry season has increased (Shen, 2010). The change in the farming system in Myanmar seems to be a cause of *S. furcifera*’s immigration to Yunnan.

In the Mekong Delta of Vietnam, in the southeastern end of the Indochina peninsula, an epidemic of *N. lugens* populations has occurred since the winter-spring rice crop in 2005–2006 (Chien et al., 2007; Hoang et al., 2011), where co-infection of two emerging viruses, *Rice grassy stunt virus* (RGSV; genus *Tenuivirus*) and *Rice ragged stunt virus* (RRSV; genus *Oryzavirus*)severely damaged more than 485,000 ha of rice production area, resulting in the loss of 828,000 tons of rice (Du et al., 2007; Cabauatan et al., 2009). The co-infection causes yellowing and light stunting of rice leaves (yellowing syndrome; Du et al., 2007). The rate of single infection with RGSV or RRSV, and the rate of co-infection with the two viruses were observed in 90, 65, and 65% of rice plants collected from 6 provinces in the delta in August 2006, respectively, whereas the rate of *N. lugens *carrying RGSV, RRSV, and both the viruses were 66, 41 and 8%, respectively (Du et al., 2007). Recently, the two genes encoded by each ambisense segment RNA3 and RNA5 of RGSV isolates from 6 provinces in southern Vietnam (5 provinces in the delta and BinhThuan province, a southeastern province near the delta) were sequenced. The results showed no relationships between the genetic diversity and the geographic distribution of the RGSV isolates, suggesting the viruliferous vector *N. lugens* migrates in southern Vietnam (Ta et al., 2013).

An escape strategy using a monitoring light trap, which shifts the timing of seeding to avoid the peak period of *N. lugens* immigration from neighboring areas, has been established in the delta to reduce viral infection (Chien et al., 2007, 2012), and campaign efforts to reduce seeding rate, fertilizer rate, and insecticide use have also decreased the density of *N. lugens* (Huan et al., 2005, 2008). Regarding the pest’s resistance to insecticides, however, the *N. lugens* population in the delta is more resistant than a population in northern Vietnam (Matsumura and Sanada-Morimura, 2010). The migration of this insecticide-resistant and viruliferous population is of much interest but is not well known. The monthly mean wind direction from 1979 to 2009 over the delta was investigated recently, and it was found that easterly winds and westerly winds dominated from October to April and from June to September, respectively (Shen, 2010; Zhai et al., 2011). Southerly or southwesterly winds that could carry planthoppers to the north of the peninsula scarcely occurred. This result suggests only a small chance that *N. lugens*’s genes flowed from the delta to the northern peninsula.

Migration in Thailand is not well known. Outbreaks of *N. lugens* have occurred in irrigated areas of central Thailand since 2009 (Chaiyawat et al., 2011). A trajectory analysis was applied to light trap data obtained in central Thailand in 2009, revealing three catch peaks: March to early April, the end of July, and November (Shen, 2010). For example, the analysis suggested that emigrants from central Thailand in July could reach Laos, central Vietnam, and Cambodia under seasonal westerly winds.

## DISCUSSION

### FLIGHT DURATION OF TROPICAL POPULATIONS

A number of migration studies of rice planthoppers in northern Vietnam, China, Japan, and Korea have been conducted, but relatively few studies have covered the Indochina peninsula and farther-western rice-producing areas such as Bangladesh and India. The previous migration studies of *N. lugens* in Thailand and southern Vietnam used flight durations of 12–36 h (Shen, 2010; Zhai et al., 2011). However, flight duration, or the flight distances of most migrating tropical populations, might be shorter than that. In the tropics, shorter migration distances, such as a few to 30 km, have been estimated by radar observations or yellow pan water-trapping in the Philippines (Perfect and Cook, 1987; Riley et al., 1987). The flight duration of *N. lugens* macropters collected in a rice field in the Philippines in a tethered flight experiment peaked at only 3–4 h (Padgham et al., 1987). Moreover, physiological characteristics such as the pre-ovipositional period and starvation tolerance of macropters of tropical *N. lugens* populations are different from those of East Asian *N. lugens *populations (Wada et al., 2007, 2009). To unveil flight durations or distances in tropical areas of Thailand, southern Vietnam, Cambodia, etc., monitoring with a tow net trap mounted to a tall pole is recommended in combination with the standard migration analysis.

### POPULATION DYNAMICS OF *S. furcifera* AND *N. lugens*

For *S. furcifera and N. lugens* in Japan, the paddy-field population is established by the overseas adult immigrants usually in July, soon after the rice transplanting (Kuno, 1968). Then *S. furcifera *generally produces two generations and the adult macropters of the second generation emigrates from the paddy fields, whereas *N. lugens* multiplies until the third generation and more, resulting in a high growth rate and causing hopperburns (Kuno, 1968). In the tropics, *S. furcifera *shows the same population dynamics as in Japan, but *N. lugens* generally produce two generations until harvest of rice, and the second generation shows a peak number (Tsurumachi, 1986). Therefore, the vector’s emigration timing may differ between in the tropics and in the temperate zone. Since rice seedling and rice plants of young stage are more susceptible to infection of RRSV than those of later stages (Tsurumachi, 1986), the Vietnamese escape strategy to escape *N. lugens*’s mass immigration, hence to avoid early viral infection, is reasonable.

### RADAR MONITORING AND RETURN MIGRATION

Entomological radar has a 40-year history and has provided much information about flying insects at altitudes from the ground (Chapman et al., 2011). Radar observation of rice planthoppers was conducted in the 1980s and early 1990s (Riley et al., 1987, 1991, 1994). Findings on observations, such as vertical group velocity of 0.2 m/s after takeoff and random heading during flight (Riley et al., 1991), have been used to model a planthopper in a migration simulation model (Furuno et al., 2005). Yang et al. (2008) developed a millimetric scanning radar (8.8 mm wavelength, 10 kw peak power, 1.2 m dish) in China and observed echoes mainly from *N. lugens* and the rice leaf roller, *Cnaphalocrocismedinalis* (Lepidoptera: Pyralidae). This radar is located in Xing’an, northeastern Guangxi, under the main migration route of rice planthoppers. An autumn return migration of *N. lugens *was analyzed by the same radar, and dense echo layers were observed at altitudes between 600 and 1,100 m above ground on the night of October 1 to 2, 2009 (Qi et al., 2010b). The migration direction was toward the southwest. Another study found a sign of collective orientation in *N. lugens* autumn migration (Jiang and Cheng, 2011), which is known as a dumbbell pattern in the radar echo (Drake and Reynolds, 2012). If collective orientation was common in *N. lugens*’s migration, the simulation model should be modified to take that into account. Further study is expected.

**FIGURE 2:**
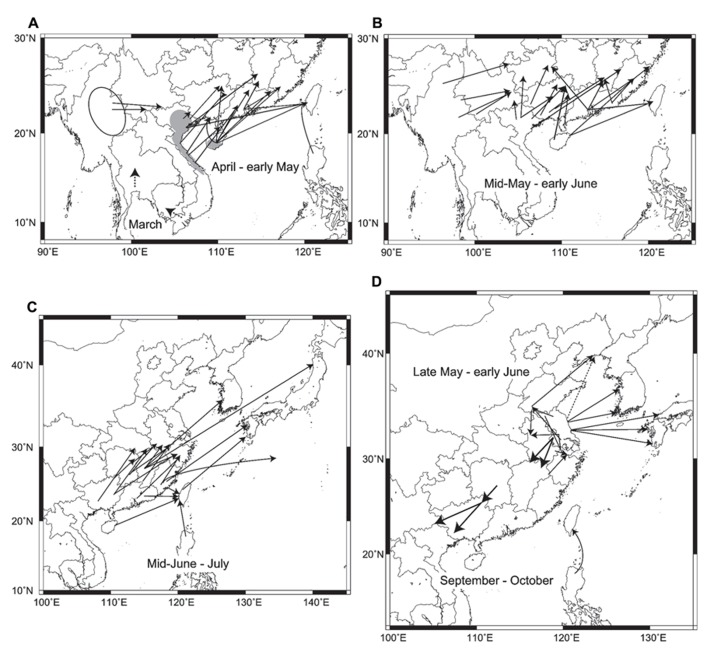
**Migration routes obtained by trajectory analyses and migration simulations:** **(A)** routes of *Sogatella furcifera* and *Nilaparvata lugens *in early season from March–April to early May. Dashed arrows in Thailand and southern Vietnam in March are hypothetical routes based on seasonal winds. Gray areas indicate overwintering areas in Vietnam and Hainan. An ellipse shows a possible source area in Myanmar. Data from Otuka et al. (2008; 2010), Qi et al. (2010a), Shen (2010), Shen et al. (2011a,b,c), Wang et al. (2011b), Zhao et al. (2011b), Zhai et al. (2011), Jiang et al. (2012), and Wu et al. (2012), **(B)** routes of *N. lugens* and *S. furcifera* from mid-May to early June. Data from the same as in **A, (C)** routes of *N. lugens* and *S. furcifera* from mid-June to July. Data from Turner et al. (1999); Hua et al. (2002), Otuka et al. (2005); Zhao et al. (2011a), Zheng et al. (2011); Diao et al. (2012), and Otuka (2012), **(D)** routes of *Laodelphax striatellus* from late May to early June, return migration routes of *N. lugens* in September to October, and a route from the Philippines to Taiwan by a typhoon in September. The dashed arrow shows the hypothetical route based on Zhou and Cheng (2012). Data from Riley et al. (1991), Otuka (2009), Qi et al. (2010b), Wang et al. (2011a); Zhang et al. (2011), Jiang et al. (2012); He et al. (2012) Otuka et al. (2012a,b), and Zhou and Cheng (2012).

In summary, the major migration routes of three rice plant- hopper species in East Asia are illustrated in **Figure 2**. This figure, which has been made for the first time from many analytical results of trajectory analysis and migration simulation, is much more specific, concrete, and accurate, including the latest situations in East Asia, than a previous summarized figure in Cheng et al. (1979).

Early immigrants of *N. lugens* and *S. furcifera* in March and April start to arrive from central Vietnam and southern Hainan in rice seedlings or paddy fields of early rice crops in southern Guangxi, Guangdong, and southern Fujian in some years (**Figure 2A**; Shen et al., 2011a,b; Wang et al., 2011b). During the same period, *S. furcifera* arrives in Yunnan from Myanmar (Shen et al., 2011c). The dashed arrows on the map of southern Vietnam and Thailand indicate possible migration in March from winter-spring crops, with unknown migration distances. By early May, rice planthoppers reach northern Guangxi, Guangdong, southern Jiangxi, Fujian, and Taiwan from northern Vietnam, Hainan, and already invaded areas in southern Guangxi and Guangdong (**Figure 2A**; Huang et al., 2010; Qi et al., 2010a; Shen et al., 2011a; Zhao et al., 2011b). The areas of invasion gradually spread northward by early June as the monsoons penetrate northward (**Figure 2B**). *N. lugens* and *S. furcifera *migrate from southern China to paddy fields in the middle and lower reaches of the Yangtze River, western Japan, and Korea from mid-June to July (**Figure 2C**). Most of the migration routes are slanted in the same direction due to southwesterly low-level jet streams, like the diagonal belt region for early migration described in Otuka et al. (2008). It was shown that overseas migration routes appear to be longer than Chinese routes over land. An estimated flight duration of 58 h (2470 km) has been reported for a migration of *S. furcifera* to northern Japan arriving on July 11, 1987 (**Figure 2C**; Otuka, 2012). A historical migration of *S. furcifera* and *N. lugens* to South Point (29°N, 135°E) over the Pacific Ocean on July 16–17, 1967 (Asahina and Turuoka, 1968) was also analyzed, and Fujian was identified as a possible source (52 h, 1770 km; Otuka, 2012). In autumn, *N. lugens* was observed by entomological radars, and trajectory analyses showed return migrations in a southwest direction (arrows pointing to the southwest in **Figure 2D**; Riley et al., 1991; Qi et al., 2010b; Jiang et al., 2012). Overseas and Chinese domestic migrations of *L. striatellus* in late May to early June are shown around Jiangsu in **Figure 2D**.

### ADDITIONAL INFORMATION TO SOURCE ESTIMATON

Trajectory analysis and migration simulation are the standard method for finding possible migration sources of rice planthoppers. Moreover, to improve this method’s accuracy, different types of additional information were combined or tested, including the data on ovarian development, genetic diversity, insecticide resistance, and trace elements in the insect’s body (Mun et al., 1999; Qi et al., 2010a; Otuka et al., 2010, 2012b; Peng et al., 2011; Fu et al., 2012; Matsumoto et al., 2013; Zheng et al., 2013). Ovarian grade in a light trap or paddy field can yield information on differences between emigrants (earliest grade) and immigrants (later grades). Genetic analyses of rice planthoppers’ populations in Asia have shown that the internal transcribed spacer (ITS) region of the ribosomal RNA gene of *S. furcifera* populations and the mitochondrial *cox1*-*trnL2*-*cox2* regions of *N. lugens* and *S. furcifera* populations did not provide molecular markers with which to discriminate Southeast Asian and East Asian populations (Mun et al., 1999; Fu et al., 2012; Matsumoto et al., 2013). This is because (1) the ITS region of *S. furcifera *is too variable to allow discrimination of local populations, and (2) rice planthoppers that migrate for long distances have well-mixed Asian populations for a long time, and the mitochondrial genetic structures of the populations reflect genetic flow over a longer period (Matsumoto et al., 2013). Thus, there has been no report thus far of a molecular marker for Asian rice planthoppers to help to determine or suggest a migration source.

Meanwhile, the insecticide resistance of both immigrant and local populations can provide good information about differences between two populations, when the two resistance levels are different in estimated source and destination areas. Such was the case when *L. striatellus* migrated from eastern China to western Japan in 2008 (Otuka et al., 2010), and when *N. lugens* migrated from the Philippines to Taiwan under typhoon-induced windy conditions in 2010 (Otuka et al., 2012b). In both cases, differences in insecticide resistance between the source and destination populations were utilized to strengthen the identification of migration sources. Recently, trace element content in *N. lugens*’s body was studied to find regional differences. In the study by Peng et al. (2011), concentrations of 23 trace elements (Mn, Mo, Cd, and etc.) in 53 samples from seven regions in southern China were determined; the samples were successfully discriminated by region as a result. In order to apply this idea to a regular migration analysis, a basic database of regional differences in trace element contents in rice planthoppers is necessary. A scientific explanation of how the difference appears based on detailed environmental factors, such as plant, soil, water, and air quality, is also expected.

## Conflict of Interest Statement

The author declares that the research was conducted in the absence of any commercial or financial relationships that could be construed as a potential conflict of interest.
